# Medial artery calcification increases neointimal hyperplasia after balloon injury

**DOI:** 10.1038/s41598-019-44668-4

**Published:** 2019-06-03

**Authors:** Andre P. Marshall, Weifeng Luo, Xue-lin Wang, Tonghui Lin, Yujun Cai, Raul J. Guzman

**Affiliations:** 1 Division of Vascular Surgery, Vanderbilt University Medical Center, Nashville, TN USA; 2 Division of Vascular and Endovascular Surgery, Beth Israel Deaconess Medical Center, Harvard Medical School, Boston, MA USA

**Keywords:** Calcification, Peripheral vascular disease, Restenosis

## Abstract

Arterial calcification predicts accelerated restenosis after angioplasty and stenting. We studied the effects of calcification on neointimal hyperplasia after balloon injury in the rat carotid. Arterial calcification was induced by subcutaneous injection of vitamin D_3_ or by adventitial application of calcium chloride. After balloon catheter injury, neointimal hyperplasia was significantly increased in rats with medial calcification compared with controls. Neointimal cell proliferation in calcified arteries as assessed by proliferating cell nuclear antigen (PCNA) staining was also higher. In calcified arteries, bone morphogenetic protein 2 (BMP-2)levels were increased at the time of injury suggesting a possible explanation for the altered responses. In vascular smooth muscle cells (SMCs) grown under calcifying conditions , stimulation with BMP-2 significantly increased cell proliferation, however, this did not occur in those grown under non-calcifying conditions. These data suggest that neointimal hyperplasia is accelerated in calcified arteries and that this may be due in part to increased BMP-2 expression in medial SMCs. Treatments aimed at inhibiting restenosis in calcified arteries may differ from those that work in uncalcified vessels.

## Introduction

Endovascular interventions have increasingly been used as first-line therapies for patients with symptomatic arterial disease^1,2^. The durability of such procedures, however, is limited by restenosis that is thought to affect at least 17% of coronary, 25% of superficial femoral, and 40% of tibial endovascular procedures within 1 year^3–5^. One factor that has increasingly been associated with accelerated restenosis rates is the amount of calcification in the arterial wall. In the coronary arteries, calcified lesions are associated with increased restenosis risk, and this remains true with drug eluting stents^6–8^. In the superficial femoral artery (SFA), calcification as seen on fluoroscopy predicts a higher failure rate and increased need for re-intervention^9^. The mechanisms that link increased calcification with restenosis, however, have not been fully explored.

Arterial calcification is generally divided into two forms that involve either the atherosclerotic intima or the media. Medial artery calcification (MAC) is most prominent in the settings of diabetes and renal disease^10^. It is related to abnormalities in bone-mineral metabolism, and the mechanisms that control it are just beginning to be understood^11^. MAC is associated with increased cardiovascular event rates including amputation^12^. The relationship between medial artery calcification and increased failure rates after endovascular interventions has not been defined.

Investigators, including us, have previously demonstrated that bone morphogenetic proteins (BMPs) and their signaling molecules can exert control over smooth muscle cell (SMC) migration, proliferation, and expression of phenotypic markers^13–18^. In calcifying arteries, medial smooth muscle cells undergo transformation to a more bone-like cell, and BMP expression is increased^19^. We thus sought to investigate whether neointimal accumulation was different after balloon injury in normal versus calcifying arteries. We further explored the effects of BMP-2 on proliferation in normal SMCs and in those undergoing phosphate-induced osteogenic transformation.

## Results

### Increased neointimal hyperplasia in vitamin D_3_-injected rats

**Figure 1:**
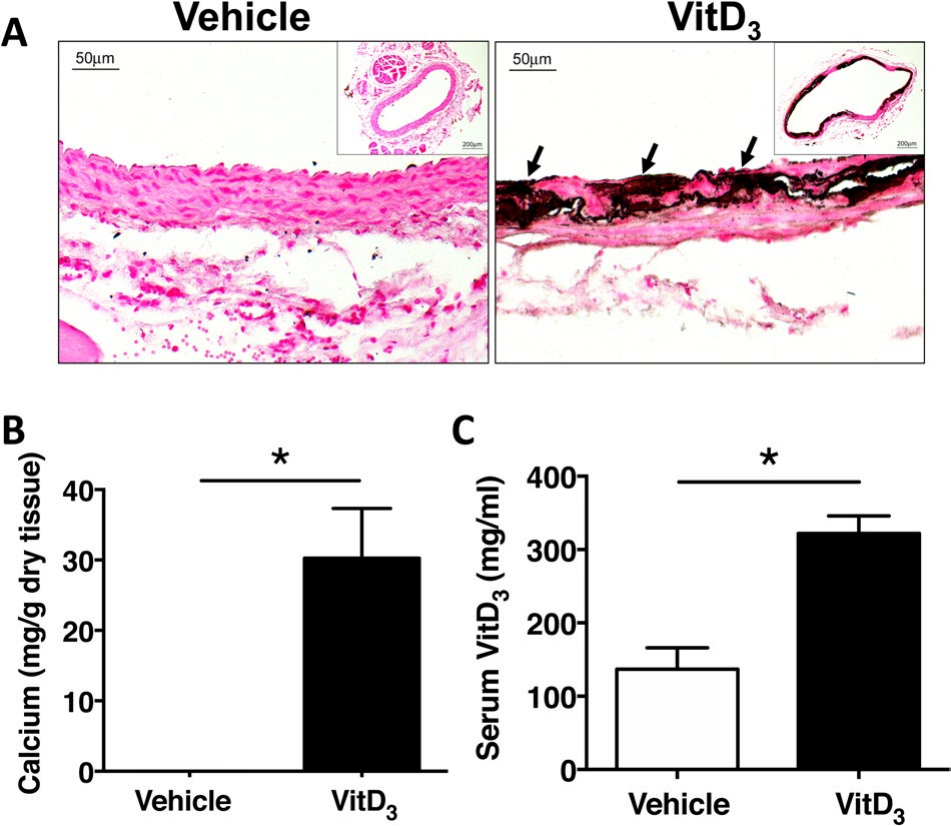
Medial calcification is induced in carotid artery after vitamin D_3_ injection. Male SD rats were injected with vehicle or 5 mg/kg vitamin D_3_ (VitD_3_) for 3 consecutive days, and carotid arteries were harvested 14 days later. (**A**) Von Kossa staining for calcium deposits. Arrows show calcium deposits in medial layer. (**B**) Calcium content was measured in the carotid arteries from vehicle (n = 7) or vitamin D_3_-injected rats (n = 11) using the o-cresolphthalein complexone method. (**C**) Vitamin D_3_ levels in serum were measured at 14 days in vehicle (n = 5) and vitamin D_3_-injected (n = 6) rats. Values are mean ± SEM. *P < 0.05.

We first assessed the effects of vitamin D_3_ administration on the rat carotid. Histologic assessment of carotid arteries at 2 weeks after vitamin D_3_ injection showed calcification in the arterial wall as demonstrated by Von Kossa staining. There was also extensive cell drop out and elastin destruction. At this time point, before balloon catheter injury, calcification was localized to the medial layer and no intimal accumulation had developed. Carotid calcium levels were significantly increased in vitamin D_3_-injected rats compared with vehicle-injected controls. (Fig. 1A,B). Vitamin D levels in serum were nearly 3-fold higher in vitamin D_3_ compared with vehicle-injected rats. (Fig. 1C).

**Figure 2:**
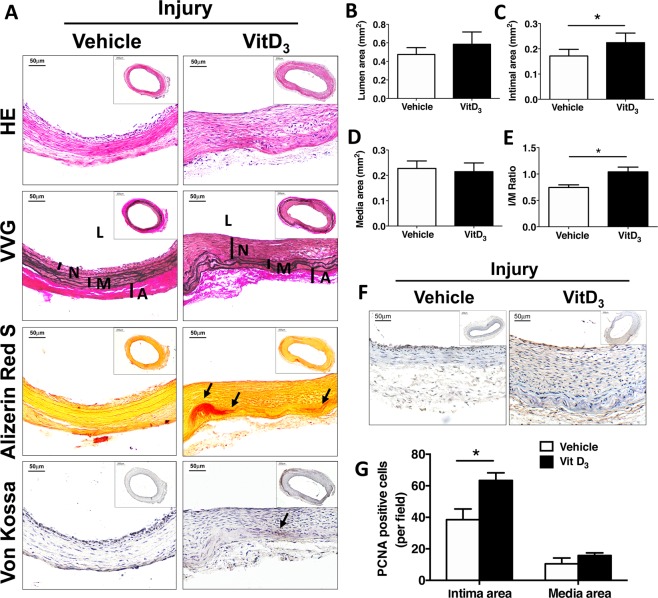
Intimal hyperplasia is increased in calcified arteries from vitamin D_3_-injected rats following balloon injury. Male SD rats were injected with vehicle or 5 mg/kg vitamin D_3_ (VitD_3_) for 3 consecutive days, and then subjected to carotid balloon injury. The carotid arteries were harvested 14 days later for histological staining and morphometric analysis. (**A**) HE staining for morphology; VVG staining for elastin fiber; Alizarin Red S and Von Kossa staining for calcium deposits. L, lumen; M, media; A, adventitial. Arrows show calcium deposits. (**B**–**E**) Morphometric analysis showed area of the lumen, intima, media, and the initma-media (I/M) ratio in rats with normal arteries (Vehicle, n = 26) and calcified carotid arteries after vitamin D_3_ injection (Vit D_3_, n = 25). (**F**,**G**) Quantified PCNA staining of normal (vehicle, n = 7) and calcified (Vit D_3_, n = 8) carotid arteries at 2 weeks after balloon injury. Values are mean ± SEM. *P < 0.05.

Balloon injury was next performed to induce neointimal formation in the left carotid artery. After three weeks, morphometric analysis of VVG stained sections showed that carotid arteries from vitamin D_3_-injected rats had significantly more neointimal hyperplasia than those from rats injected with vehicle, (1.25 ± 0.16 in vitamin D_3_ versus 0.417 ± 0.08 vehicle, *p* = 0.038, Fig. 2A–E). In response to balloon injury, the number of proliferating cells was also significantly increased in the intima of calcified arteries compared with controls (65.2 ± 5.1 versus 38.5 ± 6.8 cells/high power field, P < 0.001, Fig. F,G).

### Neointimal hyperplasia is increased in arteries with CaCl_2_-induced calcification

**Figure 3:**
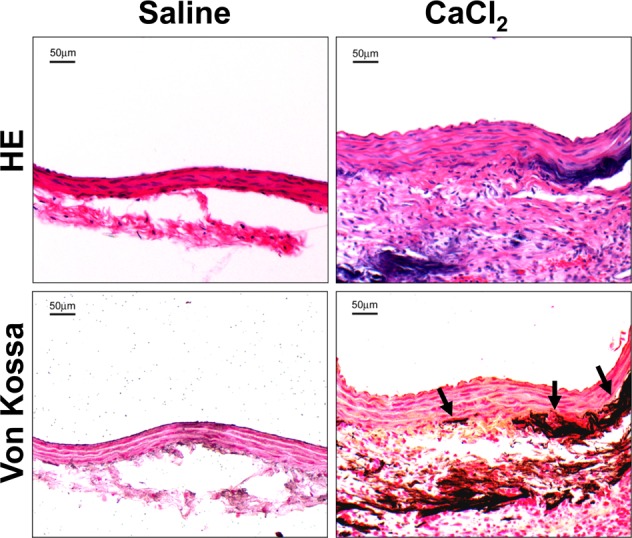
Medial calcification is induced in rat carotid arteries after application of CaCl_2_. Male SD rats was subjected to adventitial application of 0.12 M calcium chloride (CaCl_2_) in left carotid artery. One week later, carotid arteries were stained with HE. Calcium deposits were examined using Von Kossa staining. Arrows show calcium deposits.

Vitamin D_3_ is known to promote SMC proliferation *in vitro* and this is mediated in part by upregulation of VEGF^20,21^. Its levels remain elevated for at least 2 weeks after administration in rats (Fig. 1C). In order to determine whether neointimal formation is increased in calcified carotid arteries in the setting of normal Vitamin D_3_ levels, we used a second model of medial calcification induced by adventitial application of CaCl_2_^22^. In this model, calcification is induced by painting the external surface of the dissected carotid artery with a solution containing calcium chloride. Arteries treated similarly but painted with normal saline solution served as controls. Adventitial application of CaCl_2_ resulted in calcium deposition primarily localized to the outer layers of the media (Fig. 3).

**Figure 4:**
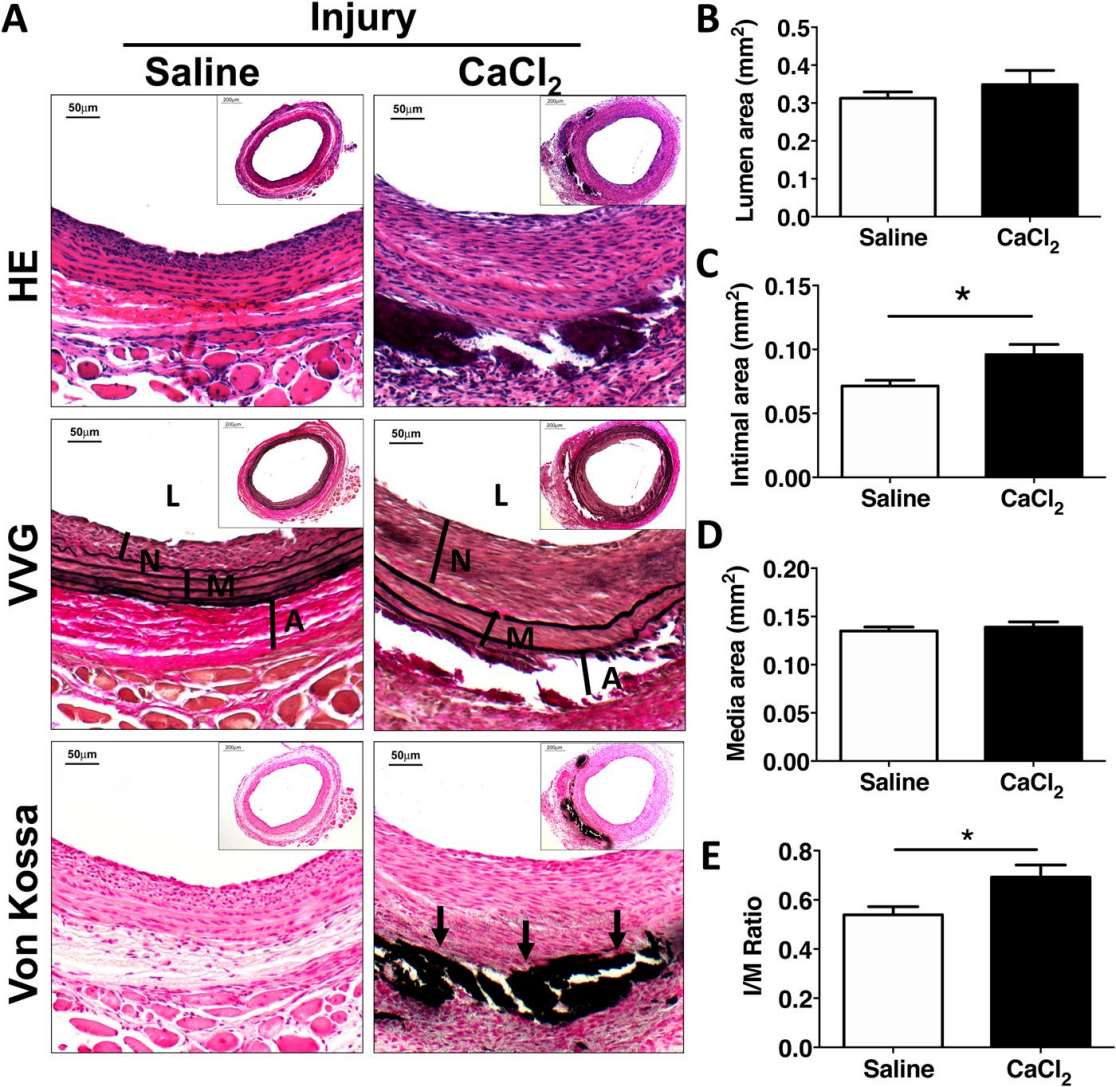
Intimal hyperplasia is increased in arteries calcified by external application of CaCl_2_ in response to balloon injury. Male SD rats were subjected to adventitial application of 0.12 M calcium chloride (CaCl_2_) to the left carotid artery and then balloon injury was performed 1 week later. After 14 days, arteries were harvested for histological staining and morphometric analysis. (**A**) HE staining for morphology; VVG staining for elastin fiber; Von Kossa staining for calcium deposits. (**B**–**E**) Morphometric analysis showing area of the lumen, intima, media, and the intima/media (I/M) ratio in rats with normal carotid arteries (saline, n = 30) and calcified arteries after application with CaCl_2_ (n = 33). L, lumen; M, media; A, adventitial. Arrows show calcium deposits. Values are mean ± SEM. *P < 0.05.

In arteries harvested 2 weeks after balloon injury, there was persistent calcification noted in the outer layers of the media (Fig. 4A). Morphometric analysis demonstrated significantly increased neointimal formation in carotids from the CaCl_2_ group than from those in the control group. (Fig. 4A–E).

### BMP-2 is increased in calcifying carotid arteries even without injury

**Figure 5:**
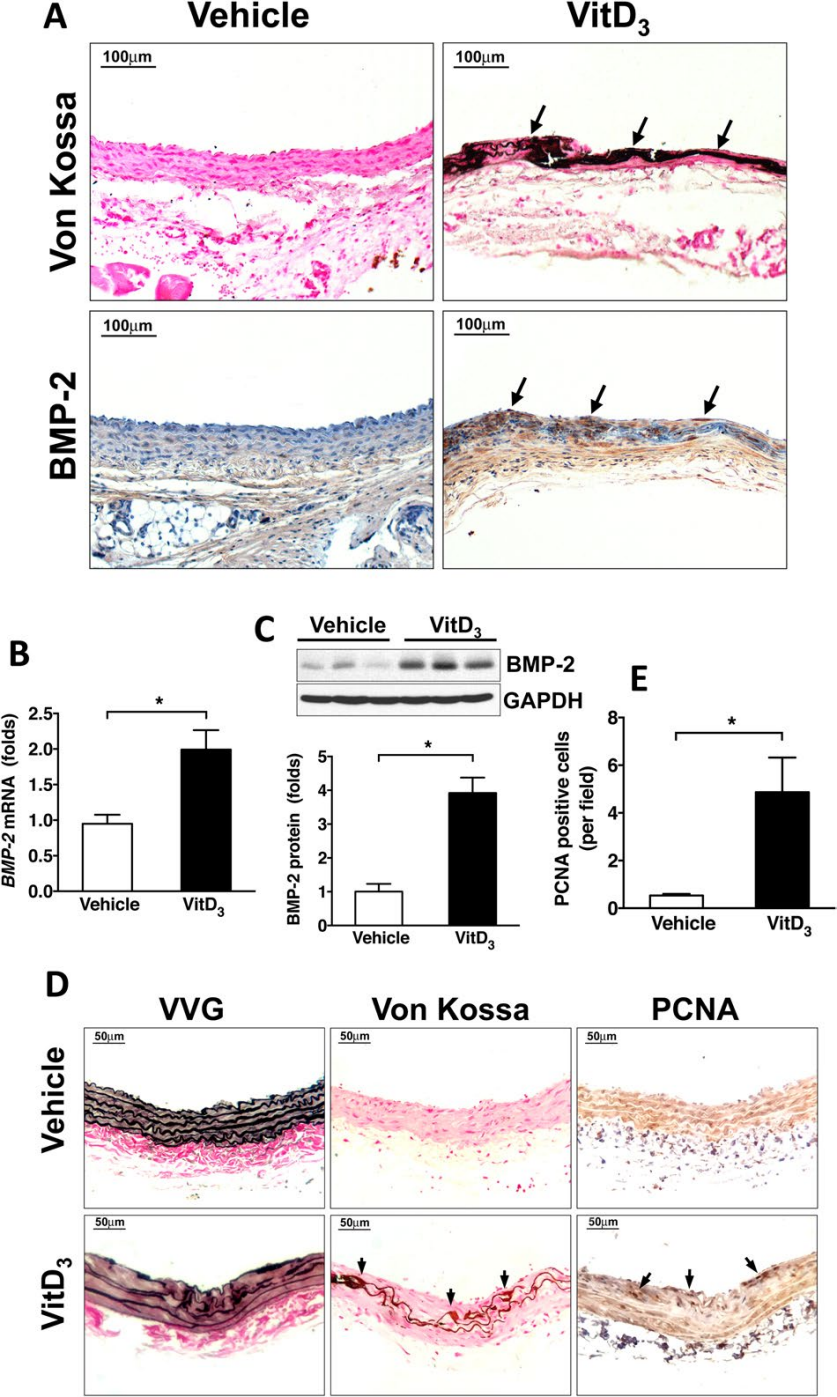
BMP-2 expression is increased in calcifying carotid arteries from vitamin D_3_-injected rats. Male SD rats were injected with vehicle or 5 mg/kg vitamin D_3_ (VitD_3_) for 3 consecutive days, and carotid arteries were harvested 14 days later. (**A**) Immunohistochemical staining showing increased BMP-2 expression in calcifying areas after vitamin D_3_ injection. (**B**) qPCR results showing increased BMP-2 mRNA levels in vitamin D_3_ injected rats. (**C**) Western blotting results showed that vitamin D_3_ injection increased BMP-2 protein levels. (**D**) Vitamin D_3_ injection increased cell proliferation in calcifying areas but did not cause neointimal hyperplasia. Neointimal hyperplasia was evaluated by vessel wall thickness. Elastin integrity was examined by VVG staining. Calcification was assessed by Von Kossa staining. Cell proliferation was determined by PCNA immunohistochemical staining. (**E**) Quantitative data showing increased PCNA-positive cells in vitamin D_3_-injected rats.

We next assessed whether calcifying carotid arteries had increased BMP expression and further, whether this could explain the increased proliferation seen in neointimal cells. In carotid arteries from rats with elevated vitamin D_3_ levels, there was significant BMP-2 staining in calcified areas whereas there was minimal baseline expression in controls (Fig. 5A). The levels of BMP-2 mRNA and protein in carotid arteries were also significantly increased in vitamin D_3_-injected rats (Fig. 5B,C). These results advance the possibility that increased BMP-2 levels seen in calcifying arteries might serve to promote SMC proliferation leading to increased neointimal accumulation. To further address if elevated BMP-2 in calcified areas could contribute to cell proliferation, we performed immunohistochemistry staining for the proliferation marker PCNA in carotid arteries from vitamin D_3_ and vehicle-treated rats. As shown in Fig. 5D, after vitamin D_3_ injection, elastic fibers were destroyed, and calcium deposits were seen along the elastic lamina. Concomitantly, cell proliferation was increased in calcifying arteries (Fig. 5D,E). However, neointima formation in these calcified but uninjured vessels was not observed.

### BMP-2 promotes proliferation in calcifying SMCs

**Figure 6:**
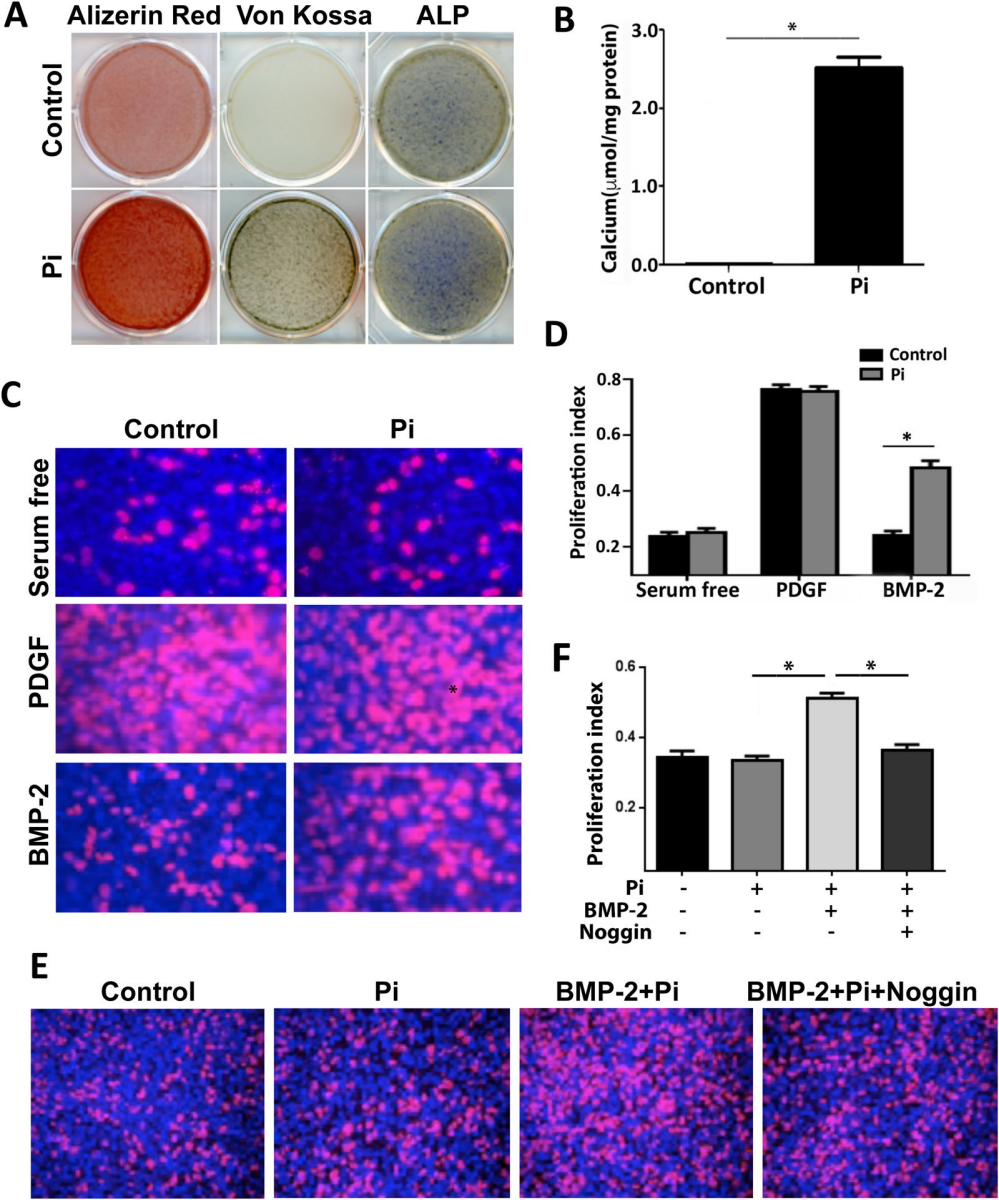
BMP-2 induces proliferation in calcifying but not control SMCs. (**A**,**B**) Inorganic phosphate (Pi) induced calcification in MOVAS cells. MOVAS cells were incubated in calcification media (5 mM sodium phosphate and 50 μg/ml sodium ascorbate) for 7 days. Calcification was determined using Alizarin Red S, Von Kossa staining, and alkaline phosphatase activity (AP) staining (**A**). Calcium content was measured using the o-cresolphthalein complexone method (**B**). (**C**,**D**) BMP-2 increased proliferation in calcifying SMCs. MOVAS cells were incubated in calcification media for 7 days, then starved for 24 h, followed by stimulation with 50 ng/ml BMP-2 or 10 ng/ml PDGF for 36 h. (**E**,**F**) Inhibition of BMP blocked BMP-2-mediated proliferation in Pi-treated SMCs. MOVAS cells were incubated in the calcification media for 7 days, then starved for 24 h, followed by stimulation with 50 ng/ml BMP-2 in the absence or presence of BMP inhibitor noggin for 36 h. Proliferation was assessed using Click-iT® EdU Cell proliferation assay. Values are mean ± SD (n = 6). *P < 0.05.

We next evaluated the effects of increased BMP-2 levels on calcifying SMCs in culture. Cells were induced to calcify by stimulation with inorganic phosphate (Pi). SMCs grown in calcification medium showed significantly elevated calcium deposition and ALP levels (Fig. 6A). We then exposed SMCs to exogenous BMP-2 added to the medium, or the positive control PDGF. We found that both calcified and non-calcified SMCs had a strong response to PDGF. In cells grown in normal medium, BMP-2 had no effect on proliferation. In calcifying cells, however, BMP-2 was able to significantly stimulate proliferation (Fig. 6C,D). To test whether the effects we observed were BMP dependent, we added the secreted BMP inhibitor Noggin that works to prevent binding of BMPs to their receptors. We found that proliferation of calcifying SMCs was reversed by addition of Noggin (Fig. 6E,F).

## Discussion

The main finding of the present work is that neointimal hyperplasia is increased in arteries with medial calcification. It was associated with increased intimal cell proliferation and medial BMP-2 expression. Cultured SMCs respond more robustly to BMP-2 when grown in calcification medium than controls cells. This suggests that upregulated BMP-2 expression in calcified arteries may promote neointimal proliferation and worsen restenosis after catheter-based interventions. Such findings may provide insight into why patients with higher arterial calcification scores have worse outcomes after endovascular interventions.

Restenosis continues to limit the durability of coronary and peripheral vascular interventions. It is related to excessive proliferation of neointimal cells leading to hemodynamically significant stenosis at sites of vascular injury. In the coronary arteries, increased target lesion revascularization (29.8% vs. 9.8%; *P* = 0.020), and restenosis (39.5% vs. 17.0%; *P* = 0.029) were seen in calcified versus non-calcified vessels^23^. In a small observational study, there was a significant positive correlation between lesion calcium score and in-stent late lumen loss at 8 months in patients following sirolimus-eluting stent implantation (r = 0.47, *p* < 0.01)^7^. Similar associations have been identified after interventions in the superficial femoral artery^9^. The mechanisms responsible for these associations, however, have not been identified.

We initially used a model of medial calcification induced by injecting high doses of vitamin D_3_ in rats. Vitamin D_3_ is a fat-soluble secosteroid with significant effects on calcium and phosphate homeostasis^24^. The model is characterized by increases in serum calcium and phosphate, and extensive ectopic calcification of elastin containing structures^25,26^. We found that intimal development after balloon injury was increased in calcified compared with non-calcified arteries. One possibility is that vitamin D_3_ directly provoked VSMC proliferation. It was previously found that rats fed a low dose of vitamin D_3_ in a concentration that did not induce medial calcification had increased intimal hyperplasia compared with controls^27^. Studies on the effects of vitamin D_3_ on SMC proliferation have yielded contradictory findings, and this is likely because its effects are dependent on the specific conditions and state of VSMCs grown in culture. For example, Mitsuhashi with others found that 1,25-(OH)_2_D_3_ increased proliferation in quiescent SMCs but decreased it in cells that were not quiescent^28^. Several investigators have shown stimulatory effects of vitamin D_3_ on SMC proliferation, and taken together, they suggest that these effects are dependent on binding to its intracellular receptor and release of VEGF via p38 activation^20,21,29–32^. Others have shown minimal or inhibitory effects related to either suppression of EGF, or through suppressed endothelin-induced SMC proliferation through inhibition of CDK2 activity and through a Cdc25A-dependent mechanism^33–35^. Moreover, It has also been shown that 1alpha, 25-(OH)_2_D_3_ induces VSMC migration independent of gene transcription via the PI3 kinase pathway^36^ Our results showed that SMC proliferation was increased in calcified and uninjured carotid arteries from vitamin D_3_-injected rats, however, there was no neointima formation observed in these vessels (Fig. 5D). This could be due to cell death occurring in arteries during calcification. By contrast, in response to injury, both SMC proliferation and neointima accumulation were significantly enhanced (Fig. 2). These data suggest that arterial injury is necessary to induce robust neointimal hyperplasia during vitamin D_3_-mediated medial calcification.

In a second series of experiments, we found that rats with carotid calcification caused by adventitial application of calcium chloride also showed increased intimal accumulation compared with control rats with similar amounts of injury but no calcification. In calcified arteries, medial SMCs showed increased expression of the bone related protein BMP-2 prior to injury. This suggests that the altered milieu present locally in calcifying arteries may predispose to increased amounts of intimal accumulation after balloon injury.

Studies on the effects of BMPs on SMC proliferation and migration have yielded inconsistent results. Initial reports showed that adenoviral BMP-2 over-expression could inhibit neointimal accumulation in a rat carotid injury model, while adding recombinant BMP-2 to cultured SMCs had minimal effect on proliferation *in vitro*^14,37^. It also decreased PDGF-induced proliferation via p21Cip1/Waf1 pathway^38^. And it has been reported that the BMP antagonist Gremlin promotes SMC migration and proliferation^17^. Indeed we have previously shown that BMP decreased PDGF-induced SMC migration but had little effect on proliferation^16^. More recently, it has been shown that BMP-2 could enhance migration and proliferation of hypoxia-induced SMCs via the Actin/CD44/MMP-2 molecular pathway^39^. Our data suggest that SMCs undergoing calcification related to increased Pi levels show increased sensitivity to BMP stimulation and that this stimulation is effectively blocked by the BMP selective inhibitor Noggin. Together these findings suggest that increased BMP expression in the media of calcifying arteries may serve as a stimulus for increased proliferation after angioplasty procedures.

In conclusion, we have shown that intimal hyperplasia is increased in two different models of medial artery calcification. Calcifying arteries show increased expression of BMP-2, and cells grown in calcifying media exhibit more BMP-induced proliferation than non-calcifying cells. These findings suggest that therapies aimed at preventing restenosis in patients with medial calcification may differ from those developed to prevent it in uncalcified vessels. Future development of such therapies may reduce restenosis and prevent the poor outcomes seen after endovascular interventions in patients with medial artery calcification.

## Materials and Methods

### Animal procedures

In order to induce medial calcification, male Sprague-Dawley rats weighing 350 to 400 g were injected subcutaneously with 5 mg/kg of Vitamin D_3_ for 3 consecutive days. Control rats received vehicle only. All animals were then allowed full access to food and water. At 2 weeks after injections, rats were anesthetized and left carotid balloon injury was performed using a 2Fr Fogarty balloon catheter as previously described^40^. At 3 weeks following injury, arterial harvest was performed. In a second series of experiments, medial calcification was induced by adventitial application of 0.12 M calcium chloride (CaCl_2_) to the exposed left carotid artery and compared with normal saline as a control. Briefly, after animals were anesthetized with Isoflurane, the left carotid artery was exposed via a neck incision and 100 μl CaCl_2_ or normal saline was applied to its adventitial surface using a sterile cotton tipped swab. Balloon injury was performed 1 week following CaCl_2_ application and arteries were harvested 2 weeks later. For all animals, perfusion fixation was performed with 10% formaldehyde and both the injured left and uninjured right carotid arteries were harvested for histological assessments. All procedures were performed under protocols approved by the Institutional Animal Care and Use Committees of Vanderbilt University Medical Center and Beth Israel Deaconess Hospital. All experiments were performed in accordance with the approved protocols.

### Morphometry, immunohistochemistry, and serum analysis

Formalin fixed carotid arteries were embedded in paraffin and cut into 5μm sections. Von Kossa staining was used to assess calcium deposits on histologic sections by incubating in 1% AgNO_3_ for one hour under UV light. Sections were then de-stained with 5% sodium thiosulfate. Nuclear fast red solution (1%) was used for counterstaining. To assess neotintimal area, Verhoeff-Van Gieson (VVG) staining was used. Briefly, sections were stained with Verhoeff working solution for 30 minutes then differentiated and de-stained with 2% ferric chloride and 5% sodium thiosulfate. Intimal and medial areas of the arterial specimens were measured using Image J on VVG stained slide sections. The intima to media ratio (I/M ratio) was calculated.

PCNA immunohistochemistry staining was performed to assess cell proliferation using a PCNA kit (Invitrogen, 931143). The Heat-Induced Epitope Retrieval (HIER) method was used for antigen retrieval. Hematoxylin was used for counterstaining. Staining was quantified by counting nuclear brown positive cells in five random fields at 40X magnification field. The PCNA positive cells were counted in both intima and media area within each field and the average was calculated. In separate groups of animals, serum vitamin D_3_ levels were measured using the Mouse/Rat 25-OH Vitamin D ELISA Assay Kit, (Eagle Biosciences) and carotid arteries were harvested for IHC staining using an anti-BMP2 antibody (Abcam, ab14933).

### Cell culture

Vascular smooth muscle cells (MOVAS, American Type Culture Collection (ATCC, Manassas, VA) were maintained in DMEM (Sigma D6429) supplemented with 10% fetal bovine serum (GIBCO), penicillin (100 U/mL)/streptomycin (0.1 mg/mL) and 0.2 mg/mL G418. Cells were cultured at 37 °C in a humidified incubator with 5% CO_2_. To induce in calcification *in vitro*, confluent SMCs were cultured in a calcification medium containing 5.0 mM sodium phosphate and 50 μg/ml sodium ascorbate. Calcification was confirmed using Alizarin Red S and Von Kossa staining. To quantify deposited calcium, cells were washed 3 times with PBS, and then extracted with 0.6 N HCl for overnight. After HCl supernatant was removed, the remaining cell layers were dissolved in 0.1 N NaOH and 0.1% SDS for quantitation of protein concentration. Calcium concentration in HCl supernatant was measured using the ο-cresolphthalein complexone method as described previously^41^. The calcium content was normalized to total protein content.

### Alkaline phosphatase staining

SMCs were cultured in a calcification medium containing 5.0 mM sodium phosphate and 50 μg/ml sodium ascorbate for 7 days. Cells were then washed twice with PBS, fixed with 10% formaldehyde for 10 min, wash with PBS, and finally stained for 30 min with staining solution (0.1 mg/ml naphthol AS-MX, 0.5% N,N-dimethyl formamide, 0.6 mg/ml fast blue BB, 0.1 M Tris-HCl, pH 8.5, 2 mM MgCl_2_). Washing with water stopped the reaction.

### Cell proliferation assay

To investigate the effects of BMP-2 on cell proliferation in calcified and non-calcified MOVAS cells, the Click-iT® EdU Cell proliferation assay (Invitrogen) was performed according to the manufacturer’s instruction. Briefly, cells were plated in 4-chamber Culture Slider (BD Falcon), and incubated in calcification media for 7–9 days, then placed in 0.2% BSA-containing media for 24 h to induce quiescence. Next, cells were treated with 50 ng/ml BMP-2 (R&D Systems) for 36 h. For positive controls, cells were stimulated with 40 ng/ml PDGF-BB (R&D Systems). In inhibition experiments, the BMP inhibitor Noggin (R&D Systems) was added to inhibit BMP-2 activity. Cells were then labeled with 15 μM EdU, fixed with 3.7% formaldehyde, and made permeable with 0.5% Triton X-100. EdU in cells was detected through the reaction with Alexa Fluor® 594 azide. Nuclei were counterstained with DAPI. The fluorescence images were separately taken at Alexa Fluor® 594 and DAPI channels on a Leica fluorescence-imaging microscope. The image of each channel was quantified by measuring the fluorescence area with Adobe Photoshop. The cell proliferation rate was presented as the ratio of EdU channel to DAPI/Hoechst channel fluorescence.

### Data analysis

Statistics were performed using GraphPad Prism. One-way ANOVA with Bonferroni’s multiple comparisons test and t-test were used for data analysis. The data were graphed and analyzed using GraphPad Prism®. Unpaired *t-test* between the IM ratio of treated and vehicle samples was used for data analysis.

## Data Availability

The datasets generated during the current study are available from the corresponding author on reasonable request.
